# Photosystems are ganging up to form megacomplexes in the dinoflagellate *Prorocentrum cordatum*

**DOI:** 10.1093/plphys/kiae093

**Published:** 2024-02-22

**Authors:** Moona Rahikainen

**Affiliations:** Assistant Features Editor, Plant Physiology, American Society of Plant Biologists; Organismal and Evolutionary Biology Research Programme, Faculty of Biological and Environmental Sciences, University of Helsinki, FI-00014 Helsinki, Finland

Phytoplankton, although small in biomass, have been estimated to account for around 45% of the global primary production (Field et al. 1998). Among phytoplankton, dinoflagellates are an ecologically successful group of unicellular eukaryotes that display high morphological diversity and varying lifestyles ranging from free-living cells to endosymbionts and parasites (Taylor et al. 2008). Photosynthetic dinoflagellates are evolutionarily distant from land plants and green algae and have structurally variable chloroplasts that originate from secondary and tertiary endosymbiosis (Taylor et al. 2008; Kalvelage et al. 2024). *Prorocentrum cordatum* is a bloom-forming marine dinoflagellate found from tropical to temperate waters (Heil et al. 2005). It is highly adaptable to changing environments and tolerant to a wide range of salinity and temperature (Dougan et al. 2023). The large *P. cordatum* haploid genome (4.15 GB) is composed of 62 chromosomes with more than 85,000 gene models (Dougan et al. 2023). The genome is characterized by a high number of dispersed duplicate genes and gene families (Dougan et al. 2023). The availability of genomic resources enables the more detailed molecular characterization of the highly adaptable *P. cordatum*.

In this issue of *Plant Physiology*, Kalvelage et al. (2024) reveal the intracellular structure of heterotrophic, free-living *P. cordatum*, characterize the organization of the photosynthetic complexes at the thylakoid membranes, and identify the proteins involved in photosynthesis. The authors used focused ion beam scanning electron microscopy to reconstruct a 3D model of *P. cordatum* cellular architecture and show that the *P. cordatum* cell encloses one large barrel-shaped chloroplast that lines the cell envelope occupying roughly 40% of the cell volume (Fig. 1A). The thylakoid membranes within do not form distinct grana structures, as in land plants, but are arranged as loose stroma-like membranes with more compact regions, as previously seen in other aquatic organisms (Grouneva et al. 2013). In *P. cordatum*, the large chloroplast encases a single mitochondrion of roughly 5% of the cell volume that forms distinct reticulate structures with close contacts to the chloroplast, suggesting a tight functional connection between the two organelles.

**Figure 1.**
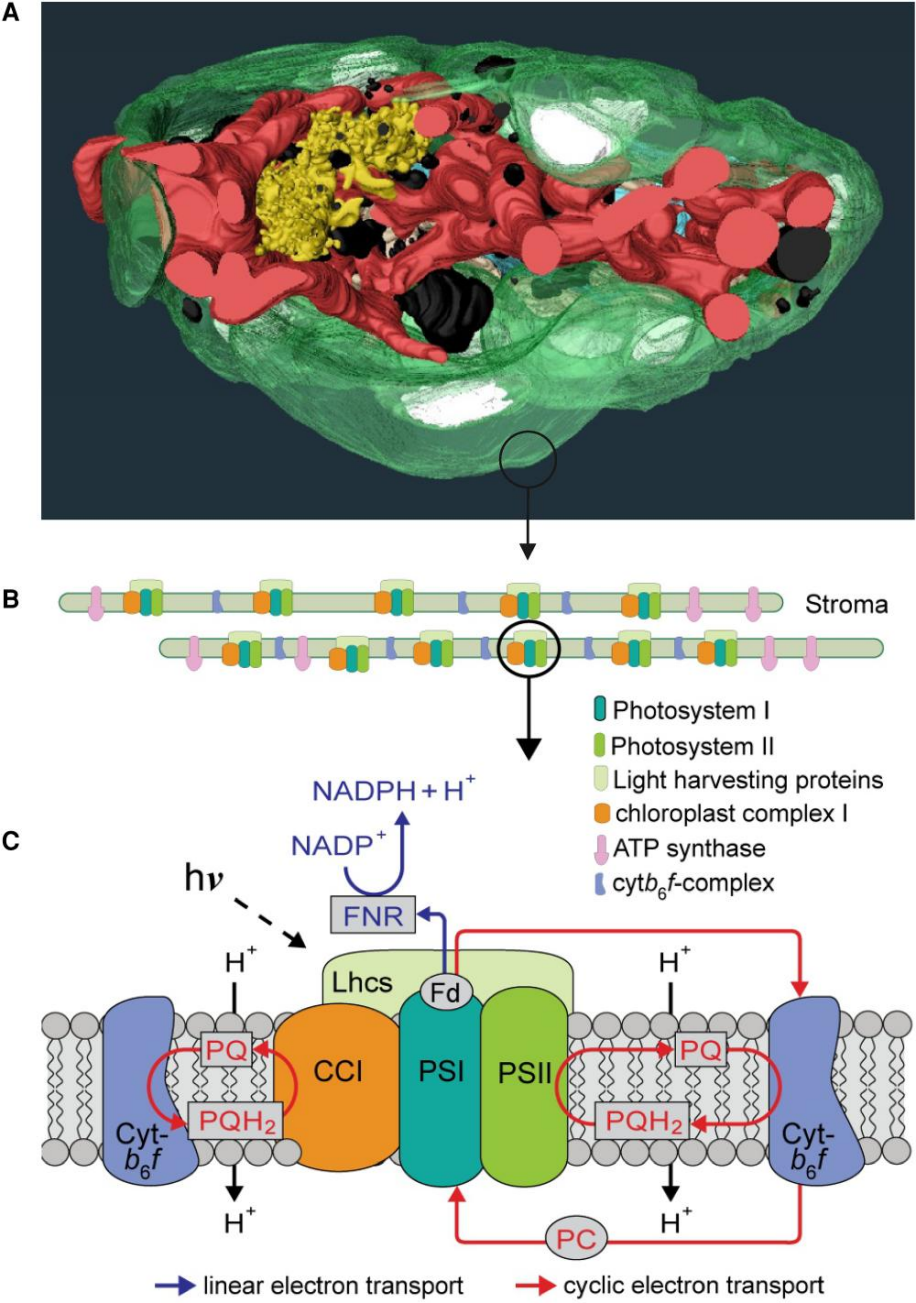
The structure of *P. cordatum* cell and organization of the photosynthetic protein complexes in the thylakoid membrane. **A)** A 3D reconstruction based on focused ion beam scanning electron microscopy micrographs showing a large barrel-shaped chloroplast (green) surrounding the reticulate mitochondrion (red), Golgi apparatus (yellow), and nucleus (blue). In addition, phosphate storage bodies (black), lipid droplets (brown), and starch granules (white) were identified. **B)** Model of the loosely arranged thylakoid membranes harboring the photosynthetic protein complexes arranged as 1.5-MDa megacomplexes. **C)** A schematic presentation of the PSI/II-LHC-CCI-megacomplex and the photosynthetic electron transfer routes. Figure was adapted from Kalvelage et al. (2024) Figs. 1 and 7.

Using blue-native and denaturing gel electrophoresis approaches combined with mass spectrometry and the available genome sequence, the authors characterized the photosynthetic machinery of *P. cordatum* and compared it to the terrestrial model plant *Arabidopsis thaliana*. In contrast to land plants, where photosystem II–light-harvesting complex II (PSII–LHCII) and photosystem I–light-harvesting complex I (PSI–LHCI) complexes are spatially separated into grana and stroma thylakoids (Rantala et al. 2017), the authors identified a large megacomplex of more than 1.5 MDa comprising both PSII and PSI, as well as chl*a-b* binding light harvesting complex proteins (LhcPs), chloroplast complex I (CCI), and other pigment-binding proteins (PBPs) (Fig. 1, B and C) (Kalvelage et al. 2024). This megacomplex contained most of the PSI and LhcPs and a notable share of PSII. According to their electrophoretic behavior, the LhcPs appeared more tightly bound to the megacomplex compared to other PBPs that were more loosely associated with the complex. Taking a closer look at the photosynthetic PBPs, Kalvelage et al. (2024) were able to identify as many as 140 predicted PBP genes in the *P. cordatum* genome and detected 83 of them with proteomic methods. The identified PBPs belonged to four categories: caroteno-chl*a–c* binding proteins (CCBPs), fucoxanthin-chl*a–c* binding proteins (FCPs), LhcPs, and green-light absorbing proteorhodopsins (GPRs). CCBPs and FCPs were the most abundant categories. Many PBPs and FCPs organized into large supercomplexes were previously observed in other microalgae and could represent an adaptation to low and fluctuating light conditions (Xu et al. 2020; Bai et al. 2021).

In land plants, electron flux between PSII and PSI under changing light conditions is balanced by state transitions where the composition of LHCs is regulated by phosphorylation of mobile LHCIIs. However, homologues of LHCII-regulating kinases and phosphatases, state transition 7/8 and protein phosphatase 1, were not identified in *P. cordatum* (Kalvelage et al. 2024). The observed PSI/II-LHC megacomplex could facilitate the efficient energy transfer and dissipation in *P. cordatum* and provide different types of regulatory mechanisms for photosynthesis. The authors hypothesize that the loosely bound PBPs could be recruited to the PSI/II-LHC megacomplex in a light-dependent manner to balance light harvesting. Notably, *P. cordatum* is missing the proton gradient regulation 5 (PGR5) and PGR5-like photosynthetic phenotype 1 (PGRL1) proteins that are associated with cyclic electron transport and balancing of ATP/NADPH ratios in chloroplast via regulation of the proton gradient that drives ATP synthesis (Grouneva et al. 2013; Kalvelage et al. 2024). Homologues of PGR5 were previously identified from diverse photosynthetic organisms, including land plants, green algae, diatoms, and cyanobacteria (Grouneva et al. 2013). In *P. cordata*, the proximity of the chloroplast and the mitochondrion may be essential for balancing the ATP/NADPH ratio in the chloroplast and for the exchange of additional metabolites between these two organelles (Bailleu et al. 2015). Altogether, the characterization of the photosynthetic complexes of *P. cordatum* by Kalvelage et al. (2024) gives a sneak-peek into the distinct differences in the organization of photosynthetic machinery between evolutionarily distant organisms. How the photosynthetic machinery of *P. cordatum* is regulated under different light regimes and environmental stresses remains a question for future research.

## Dive Curated Terms

The following phenotypic, genotypic, and functional terms are of significance to the work described in this paper:

ATP CHEBI: CHEBI:15422

PGR5 Gramene: AT2G05620
PGR5 Araport: AT2G05620

## References

[kiae093-B1] Bai T , Guo L, Xu M, Tian L. Structural diversity of photosystem I and its light-harvesting system in eukaryotic algae and plants. Front Plant Sci. 2021:12:781035. 10.3389/fpls.2021.781035 34917114 PMC8669154

[kiae093-B2] Bailleul B , Berne N, Murik O, Petroutsos D, Prihoda J, Tanaka A, Villanova V, Bligny R, Flori S, Falconet D, et al Energetic coupling between plastids and mitochondria drives CO_2_ assimilation in diatoms. Nature. 2015:524(7565):366–369. 10.1038/nature14599 26168400

[kiae093-B3] Dougan KE , Deng Z-L, Wöhlbrand L, Reuse C, Bunk B, Chen Y, Hartlich J, Hiller K, John U, Kalvelage J, et al Multi-omics analysis reveals the molecular response to heat stress in a “red tide” dinoflagellate. Genome Biol. 2023:24(1):265. 10.1186/s13059-023-03107-4 37996937 PMC10666404

[kiae093-B4] Field CB , Behrenfeld MJ, Randerson JT, Falkowski P. Primary production of the biosphere: integrating terrestrial and oceanic components. Science. 1998:281(5374):237–240. 10.1126/science.281.5374.237 9657713

[kiae093-B5] Grouneva I , Gollan PJ, Kangasjärvi S, Suorsa M, Tikkanen M, Aro EM. Phylogenetic viewpoints on regulation of light harvesting and electron transport in eukaryotic photosynthetic organisms. Planta. 2013:237(2):399–412. 10.1007/s00425-012-1744-5 22971817

[kiae093-B6] Heil CA , Glibert PM, Fan C. *Prorocentrum minimum* (Pavillard) schiller: a review of a harmful algal bloom species of growing worldwide importance. Harmful Algae. 2005:4(3):eaat4318. 10.1016/j.hal.2004.08.003

[kiae093-B7] Kalvelage J , Wöhlbrand L, Senkler J, Schumacher J, Ditz N, Bischof K, Winklhofer M, Klingl A, Braun HP, Rabus R. Conspicuous chloroplast with light harvesting-photosystem I/II megacomplex in marine *Prorocentrum cordatum*. Plant Physiol. 2024:195(1):306–325. 10.1093/plphys/kiae052 PMC11181951 38330164

[kiae093-B8] Rantala M , Tikannen M, Aro E-M. Proteomic characterization of hierarchical megacomplex formation in Arabidopsis thylakoid membrane. Plant J. 2017:92(5):951–962. 10.1111/tpj.13732 28980426

[kiae093-B9] Taylor FJR , Hoppenrath M, Saldarriaga JF. Dinoflagellate diversity and distribution. Biodivers Conserv. 2008:17(2):407–418. 10.1007/s10531-007-9258-3

[kiae093-B10] Xu C , Pi X, Huang Y, Han G, Chen X, Qin X, Huang G, Zhao S, Yang Y, Kuang T, et al Structural basis for energy transfer in a huge diatom PSI-FCPI supercomplex. Nat Commun. 2020:11(1):5081. 10.1038/s41467-020-18867-x 33033236 PMC7545214

